# Supplementary material for: The interactome of intact mitochondria by cross-linking mass spectrometry provides evidence for coexisting respiratory supercomplexes
Source: Mol Cell Proteomics. 2017 Dec 8;17(2):216–32. doi: 10.1074/mcp.RA117.000470 (PMC5795388; doi:10.1074/mcp.RA117.000470)
Supplement: Supplemental Data [file supp_RA117.000470_133922_0_supp_23978_fzffwf.zip › spectra_annotation/mito_DR_spectra_annotation/292-1-8-1-3-1.pdf]

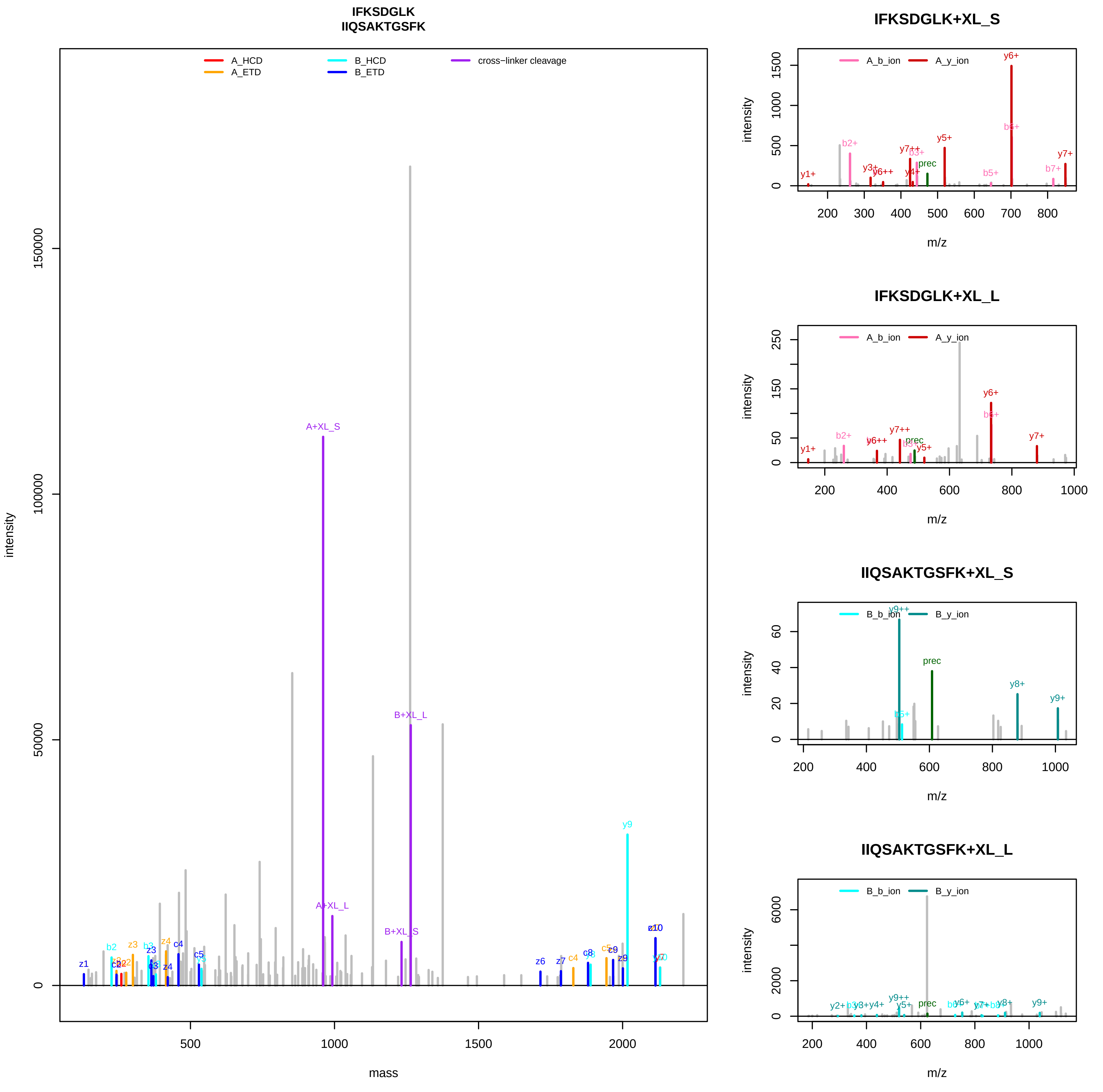
IFKSDGLK
IIQSAKTGSFK
A_HCD
A_ETD
B_HCD
B_ETD
cross−linker cleavage
A+XL_S
B+XL_L
A+XL_L
B+XL_S
y9
z10
c9
z9
c8
c5
c4
z7
z6
c5
c4
z4
z3
b2
z1
intensity
mass
0
50000
100000
150000
500
1000
1500
2000
IFKSDGLK+XL_S
A_b_ion
A_y_ion
y6+
b6+
y5+
y7++
b3+
prec
y7+
b7+
b5+
b2+
y1+
y3+
y6++
y4+
0
500
1000
1500
200
300
400
500
600
700
800
m/z
IFKSDGLK+XL_L
A_b_ion
A_y_ion
y6+
b6+
y7++
y6++
prec
y5+
b2+
y1+
y7+
0
50
150
250
200
400
600
800
1000
m/z
IIQSAKTGSFK+XL_S
B_b_ion
B_y_ion
y9++
prec
y8+
y9+
b5+
0
20
40
60
200
400
600
800
1000
m/z
IIQSAKTGSFK+XL_L
B_b_ion
B_y_ion
y9++
prec
y2+
y3+
y4+
y5+
y6+
y7+
y8+
y9+
0
2000
6000
200
400
600
800
1000
m/z
